# Integrated Scheduling Algorithm Based on Matching Game Theory in LEO Satellite Networks

**DOI:** 10.3390/s26041356

**Published:** 2026-02-20

**Authors:** Yuan Xing, Guofeng Zhao, Zhenzhen Han

**Affiliations:** 1 School of Communications and Information Engineering, Chongqing University of Posts and Telecommunications, Chongqing 400065, China; xingystudy@foxmail.com; 2 School of Cyber Security and Information Law, Chongqing University of Posts and Telecommunications, Chongqing 400065, China; hanzz@cqupt.edu.cn

**Keywords:** LEO satellite networks, time-sensitive traffic, deterministic communication, integrated scheduling, matching game theory

## Abstract

As an indispensable component of space–terrestrial integrated networks, low-Earth orbit (LEO) satellite networks are capable of providing flexible access and low delay communication services for emerging time-sensitive traffic. However, the inconsistent transmission rates between intra-satellite wired links and inter-satellite wireless links will undoubtedly result in unstable delay at the satellites. This disparity poses a challenge to ensuring deterministic communication for time-sensitive traffic. Aiming at this problem, we put forward an integrated scheduling algorithm based on matching game theory to concurrently determine the positions of wired and wireless time slots. First, we establish a theoretical model to quantify the influence of integrated scheduling on deterministic communication by elucidating the interrelationships among time-sensitive traffic, wired time slots, and wireless time slots. Second, drawing inspiration from scheduling sequences and matching game theory, the established integrated scheduling model is reformulated into a cyclic three-sided matching game model. Third, we design an integrated scheduling algorithm (ISA) to derive scheduling optimization solutions. Experimental results demonstrate that the proposed algorithm ISA outperforms existing scheduling algorithms, achieving an average delay reduction of 16.6% over all comparison algorithms.

## 1. Introduction

With the rapid growth in demand for ubiquitous and seamless communications, network technologies have gradually expanded from terrestrial to space-based. Low-Earth orbit (LEO) satellite networks will occupy an indispensable position in future space–terrestrial integrated networks (STINs) due to their wide coverage, high throughput, and low delay [1]. In recent years, mega-constellations such as Starlink, OneWeb and Telesat have provided worldwide access and seamless coverage services for terrestrial terminals [2]. Satellites carrying different payloads can cooperate with each other to provide end-to-end transmission services. Emerging time-sensitive applications such as emergency rescue, combat command, and remote control impose rigorous requirements on deterministic and real-time performances. This time-sensitive traffic needs to be delivered within a bounded low delay, meaning that in the worst case, the delay should satisfy the upper bound of the delay requirement. However, existing LEO satellite networks generally adopt the best-effort forwarding mode, which causes uncertainty in delay. Hence, one of the most challenging current problems is how to implement deterministic communication for time-sensitive traffic appearing in LEO satellite networks.

Fortunately, newly developed deterministic networking technologies such as time-sensitive networking (TSN) [3,4] and deterministic networking (DetNet) [5] can support bounded low delay, high throughput, and high reliability for both terrestrial and non-terrestrial networks. At the same time, these deterministic technologies can support the coexistence of time-sensitive and non-time-sensitive traffic. As the most mainstream deterministic technology, TSN, which originated from terrestrial ethernet, is gradually being applied to the industrial internet [6] and in-vehicle networks [7]. Wireless demand is driving the expansion of TSN to wireless networks such as WiFi and 5G [8,9]. Recently, some researchers have attempted to migrate terrestrial TSN technologies to the aerospace field [10,11] in order to improve the throughput and reliability of internal systems. By analyzing the service quality requirements on switches inside satellites, a possible TSN architecture for an intra-satellite network was designed and the feasibility of the TSN synchronization and scheduling protocols was preliminarily verified [10]. In addition, TSN ethernet technologies were designed and implemented in microlaunchers through the field-programmable gate array (FPGA) [11]. These existing studies indicate that TSN technologies can provide determinism and reliability services for an internal network of satellites.

It is well known that the transmission range of time-sensitive traffic should not be limited to internal network of satellites; rather, it is highly necessary to extend TSN technologies to wireless networks through multiple inter-satellite links. The authors of [12] presented the Space–Terrestrial Time-Sensitive Network (STTSN) architecture, in which space, aerial, and terrestrial TSN clusters are integrated. Some researchers have also attempted to solve the problem of end-to-end deterministic communication in satellite networks. To provide differentiated guarantees, multilevel cyclic queues with latency requirements were investigated in [13]. Similarly, by modifying cyclic queuing and forwarding (CQF) on a single satellite, a latency tolerance scheduling algorithm through multi-hop collaboration was proposed by [14]. Motivated by challenges of dynamic topology and limited transmission resources of satellite networks, a deterministic transmission approach was developed from aspects of routing, coding and scheduling in [15]. To achieve effective transmission of time-sensitive services in STINs, FastTS with multipath redundancy was presented in [16], with reliability assurance and delay optimization considered simultaneously. These studies all treat the satellite as a single node, but do not consider the internal network.

As can be seen from above, the time-sensitive scheduling mechanisms in intra-satellite and inter-satellite networks have been studied independently. However, the integrated scheduling on the output port of gateway has not been covered. Generally, the transmission rate of intra-satellite wired links is higher than that of inter-satellite wireless links. Inconsistent transmission rates make it impossible for data to be scheduled in a timely manner, which increases the queuing delay at the output port. In the worst case, congestion will occur at the integrated gateway inside the satellite. Thus, the bounded delay requirement of time-sensitive traffic cannot be guaranteed. Consequently, the integrated gateway becomes the bottleneck of deterministic scheduling. Existing research has not addressed this problem. Therefore, it is extremely necessary to explore an integrated scheduling algorithm in satellite networks. In this paper, an LEO constellation with wired and wireless integrated links is used as the application scenario and time-sensitive traffic is taken as the research target. To guarantee the deterministic delay experienced on the satellite, we propose an integrated scheduling algorithm based on matching game theory. The main contributions are summarized as follows:

- To accurately quantify the impact of scheduling time slots on deterministic transmission of time-sensitive traffic on the satellite, an integrated scheduling model with the optimization objective of minimizing delay is established. The established model comprehensively takes into account the relationship among time-sensitive traffic, wired time slots, and wireless time slots.
- Inspired by scheduling sequences and matching game theory, we transform the integrated scheduling model into a cyclic three-sided matching game model driven by preference lists. This transformed model simultaneously considers the preference demands of three core participating entities, namely, time-sensitive traffic, wired time slots, and wireless time slots. Each participating entity formulates the ordered ranking of decision-making preferences, which constitutes the preference list.
- To obtain scheduling optimization solutions, we design an integrated scheduling algorithm based on matching game theory. For time-sensitive traffic, the positions of the wired and wireless time slots are jointly allocated by searching for the “best” triple, and an effective solution can be derived by the proposed algorithm.

The rest of this paper is organized as follows: Section 2 presents the satellite network scenario and analyzes problems related to integrated scheduling; Section 3 establishes the network model and formalizes the integrated scheduling problem; Section 4 designs an integrated scheduling algorithm based on matching game theory; Section 5 verifies the performance through a large number of simulation experiments; finally, Section 6 summarizes the paper.

## 2. Satellite Network Scenario and Problem Statement

**Figure 1:**
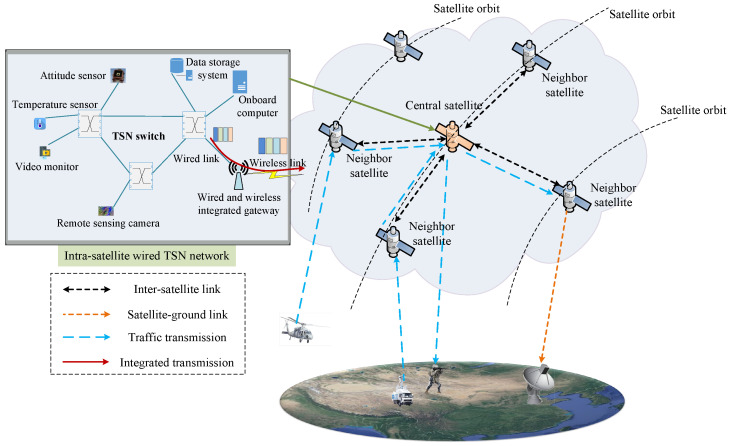
Wired and wireless integrated scheduling in an LEO constellation.

The LEO constellation scenario with wired and wireless integrated scheduling mechanism is shown in Figure 1. All devices and switches within satellites communicate with each other through wired ethernet that supports TSN technology. Laser inter-satellite links are used as the communication medium between satellites. The typical time-aware shaper (TAS) scheduling mechanism originating from the IEEE 802.1Qbv protocol [17] is adopted by the intra-satellite wired links; meanwhile, the time-division multiple access (TDMA) scheduling mechanism is utilized by the inter-satellite wireless links. Both TAS and TDMA can effectively avoid collisions by scheduling time-sensitive traffic in different time slots. As is well known, time-sensitive traffic can be transmitted over long distances through cooperation between intra-satellite wired links and inter-satellite wireless links in an LEO constellation. However, the transmission rate of wired links is usually higher than that of wireless links. Inconsistent transmission rates may increase queuing delay or even bring about congestion at the integrated gateway inside satellites. The final result is that the bounded delay requirement of time-sensitive traffic cannot be guaranteed. Fortunately, for time-sensitive traffic, the allocated intra-satellite wired time slots and inter-satellite wireless time slots will jointly determine the delay experienced on satellite. Therefore, it is very necessary to design an integrated scheduling algorithm to jointly allocate wired time slots and wireless time slots. To achieve deterministic scheduling on satellite with integrated links, the following two problems need to be resolved:

(1) How should we characterize the impact of wired and wireless integrated scheduling on the delay experienced at the satellite?

For time-sensitive traffic, the delay experienced at the satellite is determined by both wired and wireless time slots. For example, if the positions of the wired time slot and wireless time slot are both outside the transmission period of time-sensitive traffic, a scheduling timeout will occur. Ultimately, the determinism of delay is further impacted. Therefore, to achieve deterministic scheduling, it is necessary to characterize the impact of integrated scheduling on the delay experienced at the satellite.

(2) How can we achieve joint allocation of both wired and wireless time slots?

Integrated scheduling of intra-satellite wired links and inter-satellite wireless links is the key to realizing deterministic communication. Thus, for time-sensitive traffic transmitted on satellites, it is very necessary to achieve a stable forwarding delay. However, independently or randomly allocating wired time slots and wireless time slots will bring about uncertainty in the delay. Therefore, we face the issue of how to design an algorithm to realize the joint allocation of wired and wireless time slots based on the relationship among time-sensitive traffic, wired time slots, and wireless time slots.

## 3. Integrated Scheduling System Model

First, we provide an overview of the integrated wired and wireless network model in a satellite network. Second, the relationship between wired and wireless time slots is characterized by the scheduling model. Finally, the optimization objective of integrated scheduling is established.

### 3.1. Wired and Wireless Integrated Network Model

**Figure 2:**
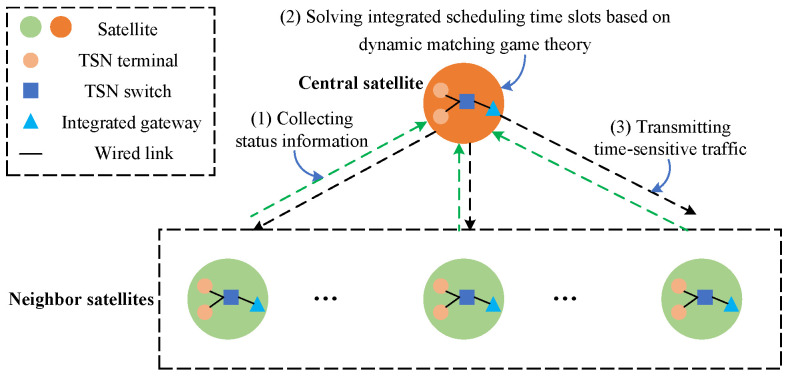
Wired and wireless integrated scheduling process.

Regarding the wired and wireless integrated LEO constellation, the network model consists of satellites v∈V and links $e\in E=\{E^{w},E^{wl}\}$. Edge $e^{w}\in E^{w}$ represents the intra-satellite wired link, while edge $e^{wl}\in E^{wl}$ denotes the inter-satellite wireless link. The set $E^{w}$ consists of terminals and switches that support TSN technology, and the topological relationship of devices within the satellite is relatively fixed. The set $E^{wl}$ is composed of edges connecting two adjacent satellites. With the periodic motion of satellites, the ISL distance between two adjacent orbital planes changes with time. However, the topology connection relationship will remain relatively stable in a period of time [18]. Thus, wired and wireless integrated scheduling can be converted into a problem of continuous scheduling within multiple adjacent time intervals. A simple integrated scheduling process is shown in Figure 2. To achieve deterministic scheduling on different satellites, the central satellite is responsible for allocating and adjusting scheduling time slots autonomously.

For ease of distinction, we define two types of time slots: topology slots and scheduling slots. A topology time slot means that the connection relationship between satellites remains unchanged within a given time. A scheduling time slot refers to the time slice used to transmit time-sensitive traffic on wired and wireless links. It is worth noting that the duration time of a topology time slot is greater than that of a scheduling time slot; this ensures that time-sensitive traffic is fully scheduled before the topology changes. The topology time slot is represented by t∈[1,T]. The total number of topology time slots is T. Let the set $I^{t}=\left\{1^{t},2^{t},\cdots ,I^{t}\right\}$ denote time-sensitive traffic in *t*, where $i^{t}\in I^{t}$; the set $J^{t}=\left\{1^{t},2^{t},\cdots ,J^{t}\right\}$ represent wired time slots in *t*, where $j^{t}\in J^{t}$; and the set $K^{t}=\left\{1^{t},2^{t},\cdots ,K^{t}\right\}$ express wireless time slots in *t*, where $k^{t}\in K^{t}$. The integrated scheduling relationship between $I^{t}$, $J^{t}$ and $K^{t}$ can be expressed as the three-sided matching $M^{t}\subseteq I^{t}\times J^{t}\times K^{t}$. For ease of reading, Table 1 provides a description of the main notation.

The variable $x_{i,j}^{t}$ indicates the assignment of the wired time slot $j^{t}$ to the time-sensitive traffic $i^{t}$. Similarly, the variable $y_{j,k}^{t}$ denotes the allocation of the wireless time slot $k^{t}$ to the wired time slot $j^{t}$. The above two categories of binary variables are defined as follows:

(1) $$x_{i,j}^{t}=\left\{\begin{array}{cc} \;1, & if\;\;j^{t}\;\;is\;\;assigned\;\;to\;\;i^{t},\\ \;0, & otherwise, \end{array}\right.$$

and

(2) $$y_{j,k}^{t}=\left\{\begin{array}{cc} \;1, & if\;\;k^{t}\;\;is\;\;assigned\;\;to\;\;j^{t},\\ \;0, & otherwise. \end{array}\right.$$

### 3.2. Wired and Wireless Integrated Scheduling Model

To intuitively display the scheduling relationship between wired time slots and wireless time slots, an integrated scheduling model for time-sensitive traffic is established. Thus, the relationship between integrated scheduling and deterministic transmission is effectively characterized via mathematical model.

#### 3.2.1. Concepts of Hyperperiod and Subperiod

**Figure 3:**
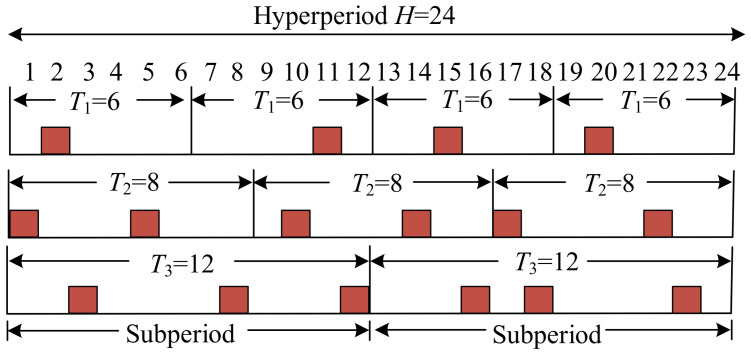
Examples of hyperperiod and subperiod.

In LEO satellite networks, time-sensitive traffic $i^{t}$ is generated at regular intervals with a fixed amount of data. For the sake of convenience, we define several parameters: the traffic generation period is represented by $T_{i^{t}}$, the data volume is indicated by $B_{i^{t}}$, and the required delay to meet the deadline is denoted as $D_{i^{t}}^{re}$. Furthermore, the properties of $i^{t}$ can be characterized by the tuple $\left\{T_{i^{t}},B_{i^{t}},D_{i^{t}}^{re}\right\}$. To facilitate effective scheduling, we introduce the concepts of hyperperiod and subperiod, as illustrated in Figure 3. The red squares in Figure 3 represent time slots occupied by time-sensitive traffic. The subperiod is designed to reduce the solution search space, while the hyperperiod is designed to avoid repetitive solution computation. The scheduling mechanism segments continuous time into multiple uniformly-sized time slots. The durations of wired time slots and wireless time slots are represented by $L_{w}$ and $L_{wl}$, respectively. The duration of the hyperperiod, denoted as $H^{t}$, is established as the least common multiple (LCM) of all individual periods $\left\{T_{i^{t}}\right\}$, specifically,

(3) $$H^{t}=LCM\left\{T_{1^{t}},T_{2^{t}},\cdots ,T_{{\left(I-1\right)}^{t}},T_{I^{t}}\right\}.$$

A subperiod is defined as a repetitive period within a hyperperiod. Hence, all time-sensitive traffic $i^{t}$ can be accommodated as integer multiples, where the number of subperiods in $H^{t}$ is defined as $M_{i^{t}}=H^{t}/T_{i^{t}}$.

Within the *m*th subperiod of $i^{t}$, the scope of wired time slot numbers is defined as the interval $\left[a_{m_{i^{t}}}^{w},b_{m_{i^{t}}}^{w}\right]$. The specific expressions are as follows:

(4) $$a_{m_{i^{t}}}^{w}=\frac{\left(m_{i^{t}}-1\right)T_{i^{t}}}{L_{w}}+1,\forall i^{t}\in I^{t},t\in \left[1,T\right]$$

and

(5) $$b_{m_{i^{t}}}^{w}=\frac{m_{i^{t}}T_{i^{t}}}{L_{w}},\forall i^{t}\in I^{t},t\in \left[1,T\right].$$

Similarly, the starting number of wireless time slots is given by

(6) $$a_{m_{i^{t}}}^{wl}=\frac{\left(m_{i^{t}}-1\right)T_{i^{t}}}{L_{wl}}+1,\forall i^{t}\in I^{t},t\in \left[1,T\right],$$

while the ending number of wireless time slot is represented as

(7) $$b_{m_{i^{t}}}^{wl}=\frac{m_{i^{t}}T_{i^{t}}}{L_{wl}},\forall i^{t}\in I^{t},t\in \left[1,T\right].$$

#### 3.2.2. Time Slot Allocation in Wired Scheduling

The intra-satellite wired link adopts the mainstream TAS scheduling mechanism. However, TAS only provides fundamental guidelines for different queues in output port through the gate control list (GCL). The specific method for determining the gate status, for example when to open or close the gate, has not been described. Given that time-sensitive traffic imposes stringent requirements regarding delay and jitter, whereas non-time-sensitive traffic does not have such constraints, the integrated scheduling design primarily focuses on the GCL designated for time-sensitive traffic.

The variable $x_{i,j}^{t}$ determines the allocation of wired time slots and takes values in {0,1}. Specifically, $x_{i,j}^{t}=1$ signifies that the gate is open, allowing scheduling of $i^{t}$ in $j^{t}$. Conversely, if $x_{i,j}^{t}=0$, the gate remains closed, and scheduling is prohibited. To avoid conflicts, each $j^{t}$ can only be assigned to one $i^{t}$.

(8) $$\sum_{i^{t}\in I^{t}}x_{i,j}^{t}\leq 1,\forall j^{t}\in J^{t},t\in \left[1,T\right]$$

Furthermore, the total number of assigned wired time slots must not surpass the overall number of available wired time slots $J^{t}$.

(9) $$\sum_{i^{t}\in I^{t}}\sum_{j^{t}\in J^{t}}x_{i,j}^{t}\leq J^{t},\forall t\in \left[1,T\right]$$

Let $s_{m_{i^{t}}}$ represent the number of wired time slots allocated to $i^{t}$ in the *m*th subperiod, which can be expressed as

(10) $$s_{m_{i^{t}}}=\sum_{j^{t}=a_{m_{i^{t}}}^{w}}^{b_{m_{i^{t}}}^{w}}x_{i,j}^{t},\forall i^{t}\in I^{t},t\in \left[1,T\right].$$

To guarantee that $i^{t}$ is transmitted with low delay and that the generated traffic does not accumulate, it is necessary to complete scheduling within the subperiod. Referring to Equations (9) and (10), we derive the following:

(11) $$\sum_{i^{t}=1}^{I^{t}}\sum_{m_{i^{t}}=1}^{M_{i^{t}}}s_{m_{i^{t}}}=\sum_{i^{t}=1}^{I^{t}}\sum_{j^{t}=1}^{J^{t}}x_{i,j}^{t}=\sum_{i^{t}=1}^{I^{t}}\sum_{m_{i^{t}}=1}^{M_{i^{t}}}\sum_{j^{t}=a_{m_{i^{t}}}^{w}}^{b_{m_{i^{t}}}^{w}}x_{i,j}^{t}\leq J^{t},\forall t\in \left[1,T\right].$$

#### 3.2.3. Time Slot Allocation in Wireless Scheduling

To prevent collisions during wireless scheduling, it is imperative that each wireless time slot $k^{t}$ be assigned exclusively to a single wired time slot $j^{t}$.

(12) $$\sum_{j^{t}\in J^{t}}y_{j,k}^{t}\leq 1,\forall k^{t}\in K^{t},t\in \left[1,T\right]$$

Moreover, the cumulative quantity of wireless time slots assigned for wired time slots must not surpass the total number $K^{t}$.

(13) $$\sum_{j^{t}\in J^{t}}\sum_{k^{t}\in K^{t}}y_{j,k}^{t}\leq K^{t},\forall t\in \left[1,T\right]$$

For wireless link $e^{wl}$, the transmission rate is denoted as $R_{e^{wl}}^{t}$, which is influenced by link conditions. The specific mathematical expression of $R_{e^{wl}}^{t}$ can be found in [19]. For wired link $e^{w}$, the transmission rate is expressed as $R_{e^{w}}$. Generally, $R_{e^{w}}$ surpasses $R_{e^{wl}}^{t}$, which may lead to congestion at the integrated gateway. To guarantee that the traffic transmitted during a wired time slot is fully accommodated by a corresponding wireless time slot, the following configuration relationship should be satisfied:

(14) $$R_{e^{wl}}^{t}L_{wl}\geq R_{e^{w}}L_{w}.$$

To attain integrated scheduling for time-sensitive traffic $i^{t}$, the wired time slot must be scheduled earlier than the wireless time slot. Therefore, the positional relationship between the allocated wired time slot and wireless time slot is represented as follows:

(15) $$j^{t}x_{i,j}^{t}L_{w}\leq \left(k^{t}-1\right)y_{j,k}^{t}L_{wl},\forall i^{t}\in I^{t},j^{t}\in J^{t},k^{t}\in K^{t},t\in \left[1,T\right].$$

#### 3.2.4. Wired and Wireless Integrated Scheduling Modeling

**Figure 4:**
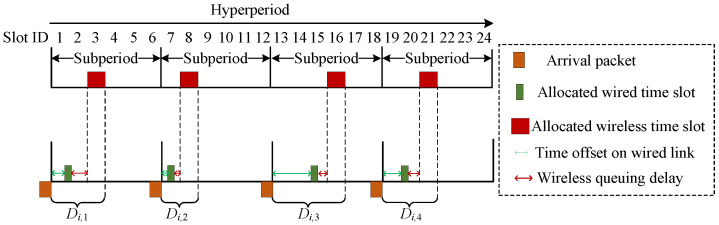
Wired and wireless integrated scheduling diagram.

By integrated scheduling, we mean the phenomenon by which wired and wireless time slots are allocated in conjunction. Figure 4 presents a schematic diagram illustrating the components of delay under integrated scheduling. Specifically, the delay experienced by time-sensitive traffic is primarily influenced by periodic arrival characteristics, transmission rates, and scheduling time slots. The time of arrival (ToA) of time-sensitive traffic $i^{t}$ in its *m*th subperiod is mathematically expressed as

(16) $$ToA_{m_{i^{t}}}=t_{\mathrm{start}}^{t}+\left(m_{i^{t}}-1\right)T_{i^{t}},\forall i^{t}\in I^{t},t\in \left[1,T\right].$$

The time $t_{\mathrm{start}}^{t}$ is the absolute start time of topology time slot *t*.

For time-sensitive traffic $i^{t}$ in the *m*th subperiod, the delay determined by integrated scheduling can be computed as follows:

(17) $$D_{m_{i^{t}}}=\delta_{m_{i^{t}}}+D_{m_{i^{t}}}^{Tran}+D_{m_{i^{t}}}^{Que\_wl},\forall i^{t}\in I^{t},t\in \left[1,T\right],$$

where $\delta_{m_{i^{t}}}$ is the time offset on the wired link, $D_{m_{i^{t}}}^{Tran}$ is the transmission delay, and $D_{m_{i^{t}}}^{Que\_wl}$ is the wireless queuing delay.

In addition, $\delta_{m_{i^{t}}}$ is characterized as the temporal delay that occurs between the moment of traffic generation and the corresponding position of the assigned wired time slot, which is represented as

(18) $$\delta_{m_{i^{t}}}=\sum_{j^{t}=a_{m_{i^{t}}}^{w}}^{b_{m_{i^{t}}}^{w}}\left(j^{t}-a_{m_{i^{t}}}^{w}\right)x_{i,j}^{t}L_{w},\forall i^{t}\in I^{t},t\in \left[1,T\right].$$

Here, $D_{m_{i^{t}}}^{Tran}$ is comprised of two components: the transmission delay experienced on the wired link, denoted as $D_{m_{i^{t}}}^{Tran\_w}$, and the transmission delay experienced on wireless link, represented as $D_{m_{i^{t}}}^{Tran\_wl}$.

(19) $$D_{m_{i^{t}}}^{Tran}=D_{m_{i^{t}}}^{Tran\_w}+D_{m_{i^{t}}}^{Tran\_wl}=\frac{B_{i^{t}}}{R_{e^{w}}}+\frac{B_{i^{t}}}{R_{e^{wl}}^{t}},\forall i^{t}\in I^{t},t\in \left[1,T\right]$$

In the above equation, $D_{m_{i^{t}}}^{Que\_wl}$ represents the queuing delay before the traffic is scheduled by the wireless time slot but completed by the wired time slot, and is defined as the temporal difference between the finishing of wired scheduling and the beginning of wireless scheduling. Within the integrated scheduling design, the constraint shown in (15) should be satisfied simultaneously. In accordance with the positional relationship between allocated wired time slot $j^{t}$ and wireless time slot $k^{t}$, we can express $D_{m_{i^{t}}}^{Que\_wl}$ as

(20) $$D_{m_{i^{t}}}^{Que\_wl}=\sum_{k^{t}=a_{m_{i^{t}}}^{wl}}^{b_{m_{i^{t}}}^{wl}}\sum_{j^{t}=a_{m_{i^{t}}}^{w}}^{b_{m_{i^{t}}}^{w}}\left[\left(k^{t}-1\right)L_{wl}-j^{t}L_{w}\right]x_{i,j}^{t}y_{j,k}^{t},\forall i^{t}\in I^{t},t\in \left[1,T\right].$$

### 3.3. Problem Formulation for Integrated Scheduling

As presented in (21), we establish the optimization objective to minimize the delay associated with time-sensitive traffic. Constraint (22) indicates whether $j^{t}$ is allocated to $i^{t}$. Constraint (23) indicates whether $k^{t}$ is allocated to $j^{t}$. Constraint (24) indicates that each $j^{t}$ can only be allocated to one $i^{t}$. Constraint (25) means that the aggregate number of wired time slots assigned to all time-sensitive traffic must not surpass the total number of available wired time slots $J^{t}$. Constraint (26) indicates that each $k^{t}$ can be assigned to only one $j^{t}$. Constraint (27) specifies that the total number of wireless time slots allocated to all wired time slots cannot exceed the total number of wireless time slots $K^{t}$. Constraint (28) shows that the scheduling of a wired time slot must precede that of a wireless time slot. Constraint (29) means that the delay associated with integrated scheduling must not exceed the predetermined delay requirement.

(21) $$\begin{array}{cc} \min\; & D_{m_{i^{t}}},\;\forall i^{t}\in I^{t},t\in \left[1,T\right], \end{array}$$

(22) $$\begin{array}{cc} s.t.\; & x_{i,j}^{t}\in \left\{0,1\right\},\forall i^{t}\in I^{t},j^{t}\in J^{t},t\in \left[1,T\right], \end{array}$$

(23) $$\begin{array}{cc} \; & y_{j,k}^{t}\in \left\{0,1\right\},\forall j^{t}\in J^{t},k^{t}\in K^{t},t\in \left[1,T\right], \end{array}$$

(24) $$\begin{array}{cc} \; & \sum_{i^{t}\in I^{t}}x_{i,j}^{t}\leq 1,\forall j^{t}\in J^{t},t\in \left[1,T\right], \end{array}$$

(25) $$\begin{array}{cc} \; & \sum_{i^{t}\in I^{t}}\sum_{j^{t}\in J^{t}}x_{i,j}^{t}\leq J^{t},\forall t\in \left[1,T\right], \end{array}$$

(26) $$\begin{array}{cc} \; & \sum_{j^{t}\in J^{t}}y_{j,k}^{t}\leq 1,\forall k^{t}\in K^{t},t\in \left[1,T\right], \end{array}$$

(27) $$\begin{array}{cc} \; & \sum_{j^{t}\in J^{t}}\sum_{k^{t}\in K^{t}}y_{j,k}^{t}\leq K^{t},\forall t\in \left[1,T\right], \end{array}$$

(28) $$\begin{array}{cc} \; & j^{t}x_{i,j}^{t}L_{w}\leq \left(k^{t}-1\right)y_{j,k}^{t}L_{wl},\forall i^{t}\in I^{t},j^{t}\in J^{t},k^{t}\in K^{t},t\in \left[1,T\right], \end{array}$$

(29) $$\begin{array}{cc} \; & D_{m_{i^{t}}}\leq D_{i^{t}}^{re},\forall i^{t}\in I^{t},t\in \left[1,T\right]. \end{array}$$

The decision variables $x_{i,j}^{t}$ and $y_{j,k}^{t}$ indicate that the integrated scheduling problem presented above is classified as a 0–1 integer programming problem, which is recognized as NP-complete. However, the purpose of integrated scheduling is to obtain feasible solutions that satisfy delay constraints. Inspired by scheduling sequences and allocation relationships, we introduce matching game theory to address integrated scheduling problem. The algorithm informed by matching game theory is capable of generating suboptimal solutions while also accommodating the preferences of each involved entity.

## 4. Integrated Scheduling Algorithm Based on Matching Game Theory

According to types of participating entities, the wired and wireless integrated scheduling model mentioned above can be transformed into a cyclic three-sided matching game model. Moving forward, we design an integrated scheduling algorithm based on matching game theory.

### 4.1. Three-Sided Matching Game Model

A three-sided matching game can effectively represent the relationship between participating entities, for example the three-sided relationship composed of “man–woman–dog” in the real world [20]. The main feature of the matching game is that each class of entity only has a preference list for another class of entity [20]. Therefore, in the integrated scheduling model, time-sensitive traffic only has a preference list $P_{i^{t}}$ for wired time slots, wired time slots only have a preference list $P_{j^{t}}$ for wireless time slots, and wireless time slots only have a preference list $P_{k^{t}}$ for time-sensitive traffic. For time-sensitive traffic $I^{t}$, wired time slot $J^{t}$, and wireless time slot $K^{t}$, all the possible triples are expressed as $I^{t}\times J^{t}\times K^{t}=T^{t}$. The three-sided matching set is denoted with $M^{t}\subseteq T^{t}$. For the matching triple $\left(i^{t},j^{t},k^{t}\right)\in M^{t}$, $M^{t}\left(i^{t}\right)=j^{t}$ means that wired time slot $j^{t}\in J^{t}$ is only accepted by time-sensitive traffic $i^{t}\in I^{t}$, while $M^{t}\left(j^{t}\right)=k^{t}$ implies that wireless time slot $k^{t}\in K^{t}$ is only accepted by wired time slot $j^{t}\in J^{t}$ and $M^{t}\left(k^{t}\right)=i^{t}$ indicates that time-sensitive traffic $i^{t}\in I^{t}$ is only accepted by wireless time slot $k^{t}\in K^{t}$.

The ultimate purpose of the three-sided matching game is to discover a stable matching set under preference lists. The blocking triple can effectively indicate whether the three-sided matching game has reached a stable matching. The specific definition of a blocking triple is expressed below.

**Definition 1.**  *The triple $\left(i^{t},j^{t},k^{t}\right)\notin M^{t}$ but $\left(i^{t},j^{t},k^{t}\right)\in T^{t}$ is a blocking triple if the following set is satisfied:*

(30) $$\begin{array}{c} \{M^{t}\left(i^{t}\right)=⌀\vee j^{t}{≻}_{i^{t}}M^{t}\left(i^{t}\right)\}\wedge \\ \{M^{t}\left(j^{t}\right)=⌀\vee k^{t}{≻}_{j^{t}}M^{t}\left(j^{t}\right)\}\wedge \\ \{M^{t}\left(k^{t}\right)=⌀\vee i^{t}{≻}_{k^{t}}M^{t}\left(k^{t}\right)\}, \end{array}$$

*where $j^{t}{≻}_{i^{t}}M^{t}\left(i^{t}\right)$ means that time-sensitive traffic $i^{t}$ prefers wired time slot $j^{t}$ to its current matched wired time slot $M^{t}\left(i^{t}\right)$, $k^{t}{≻}_{j^{t}}M^{t}\left(j^{t}\right)$ indicates that wired time slot $j^{t}$ prefers wireless time slot $k^{t}$ to its current matched wireless time slot $M^{t}\left(j^{t}\right)$, and $i^{t}{≻}_{k^{t}}M^{t}\left(k^{t}\right)$ indicates that wireless time slot $k^{t}$ prefers time-sensitive traffic $i^{t}$ to its current matched time-sensitive traffic $M^{t}\left(k^{t}\right)$. If there are no blocking triples, the three-sided matching $M^{t}$ is considered stable.*

**Figure 5:**
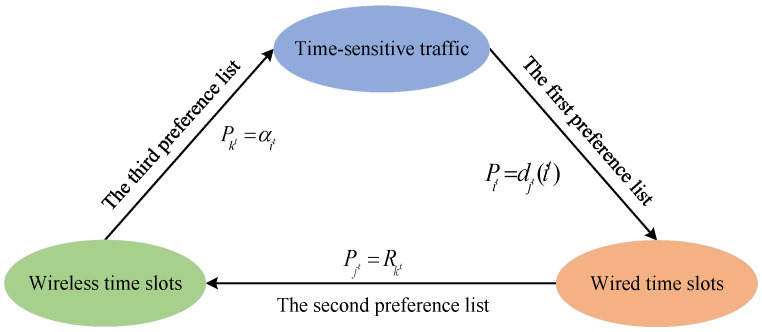
Cyclic three-sided matching game model for time-sensitive traffic.

However, the traditional three-sided matching game model lacks a representation of relationships among different preference lists, which makes it difficult to solve. Inspired by scheduling sequences, we transform the integrated scheduling model into a cyclic three-sided matching game model for time-sensitive traffic, as shown in Figure 5. In circular preference lists, $P_{i^{t}}$, $P_{j^{t}}$, and $P_{k^{t}}$ are set as the first, second, and third preference lists, respectively. Different matching entities are constrained through corresponding preference lists. Specifically, the preference lists $P_{i^{t}}$, $P_{j^{t}}$, and $P_{k^{t}}$ are formed as follows: the preference list $P_{i^{t}}$ of time-sensitive traffic for wired time slots is sorted according to the wired scheduling delay $d_{j^{t}}\left(i^{t}\right)$, that is, time-sensitive traffic prefers wired time slots with lower delay; the preference list $P_{j^{t}}$ of wired time slots for wireless time slots is sorted according to the wireless link transmission rate $R_{k^{t}}$, meaning that wired time slots prefer wireless time slots with higher transmission rates; and the preference list $P_{k^{t}}$ of wireless time slots for time-sensitive traffic is sorted in accordance with priority $\alpha_{i^{t}}$, that is, wireless time slots preferentially select time-sensitive traffic with high priority for scheduling.

Before providing the details of the designed scheduling algorithm, the necessary sets are defined as follows:

(31) $$A^{+1}\left(M^{t},i^{t}\right)=\left\{j^{t}\left|j^{t}{≻}_{i^{t}}M^{t}\left(i^{t}\right),j^{t}\in P_{i^{t}}\right.\right\}.$$

For time-sensitive traffic $i^{t}$, there exists a set of wired time slots that are more preferred than $i^{t}$’s existing matching $M^{t}\left(i^{t}\right)$:

(32) $$A^{+1}\left(M^{t},j^{t}\right)=\left\{k^{t}\left|k^{t}{≻}_{j^{t}}M^{t}\left(j^{t}\right),k^{t}\in P_{j^{t}}\right.\right\}.$$

For wired time slot $j^{t}$, there exists a set of wireless time slots that are preferred over $j^{t}$’s current matching $M^{t}\left(j^{t}\right)$:

(33) $$A^{-1}\left(M^{t},i^{t}\right)=\left\{k^{t}\left|k^{t}\in K^{t},i^{t}\in P_{k^{t}},N\left(M^{t},k^{t}\right)\leq N_{k^{t}}\right.\right\}$$

which represents the set of wireless time slots that can still accommodate time-sensitive traffic:

(34) $$A^{-2}\left(M^{t},i^{t}\right)=\left\{j^{t}\left|j^{t}\in J^{t},A^{+1}\left(M^{t},j^{t}\right)\cap A^{-1}\left(M^{t},i^{t}\right)\neq 0\right.\right\}.$$

For wired time slot $j^{t}$, there is a wireless time slot $k^{t}$ that is more preferred than $j^{t}$’s existing matching $M^{t}\left(j^{t}\right)$ while still being capable of accommodating the time-sensitive traffic $i^{t}$.

### 4.2. Integrated Scheduling Algorithm

By combining the above cyclic three-sided matching game model, we design an integrated scheduling algorithm (ISA) that can realize joint allocation of wired and wireless time slots. The basic idea of the ISA is to search for the “best” triple through circular preference lists. For each time-sensitive traffic instance, it selects the best wired time slot satisfying the requirements; then, the selected wired time slot chooses the best wireless time slot that meets its requirements. When the time-sensitive traffic completes the scheduling request, a stable matching triple between time-sensitive traffic and scheduling time slots is obtained. At last, these generated triples are added to the matching set $M^{t}$.

In light of these preference definitions, Algorithm 1 describes the detailed procedures of the proposed ISA. Here, we introduce the indicators “flag” to guide the execution of the algorithm and Head(X,z) to represent the element in *X* which has the highest priority in preference list of *z*. For the proposed algorithm, the input consists of the participating entities $I^{t}$, $J^{t}$, and $K^{t}$. The output is the matching set $M^{t}$ containing integrated scheduling results. The algorithm mainly consists of two stages: the initialization stage (lines 1∼3), and the matching game stage (lines 4∼25). In the initialization stage, the preference lists $P_{i^{t}}$, $P_{j^{t}}$ and $P_{k^{t}}$ are created according to the known preference information. The set $M^{t}$ is initialized to the empty set ⌀ and the “flag” indicator is set to one. To satisfy the deadline delay requirement, the time slot allocation order for time-sensitive traffic is based on priority $\alpha_{i^{t}}$ in descending order. In the matching game stage, for each time-sensitive traffic $i^{t}$, a better wired time slot $j^{\prime t}$ is discovered in steps 7∼9. If there exists such a wired time slot, a better wireless time slot $k^{\prime t}$ for $j^{\prime t}$ is searched in steps 10∼11. Next, the capacity of time-sensitive traffic $i^{t}$ is checked. If $N\left(M^{t},i^{t}\right)==I^{t}$, then the worst matching triple $\left(i^{t},M^{t}\left(i^{t}\right),M^{t}\left(M^{t}\left(i^{t}\right)\right)\right)$ is deleted (lines 12∼16). Similarly, the capacity of wired time slot $j^{\prime t}$ is checked and dealt with in steps 17∼20. Then, the newly generated triple $\left(i^{t},j^{\prime t},k^{\prime t}\right)$ is added to the set $M^{t}$. The matching process is iterated until reaching maximum cardinality $\left|M^{t}\right|$. At last, a stable matching set $M^{t}$ in topology time slot *t* is acquired.
**Algorithm 1** Integrated Scheduling Algorithm**Input:** $I^{t}$, $J^{t}$, $K^{t}$;**Output:** $M^{t}$;  1:*Initialization*: Create the preference lists $P_{i^{t}}$, $P_{j^{t}}$ and $P_{k^{t}}$;  2:Set $M^{t}=⌀$ and flag = 1;  3:Sort time-sensitive traffic $i^{t}$ in descending order of priority $\alpha_{i^{t}}$;  4:**while** flag == 1 **do**  5:   Set flag = 0;  6:   **for** each time-sensitive traffic $i^{t}\in I^{t}$ **do**  7:      $J^{\prime t}=A^{+1}\left(M^{t},i^{t}\right)\cap A^{-2}\left(M^{t},i^{t}\right)$;  8:      **if** $J^{\prime t}\neq ⌀$ **then**  9:           $j^{\prime t}=Head\left(J^{\prime t},i^{t}\right)$;10:           $K^{\prime t}=A^{+1}\left(M^{t},j^{t}\right)\cap A^{-1}\left(M^{t},i^{t}\right)$;11:           $k^{\prime t}=Head\left(K^{\prime t},j^{t}\right)$;12:           **if** $N\left(M^{t},i^{t}\right)==I^{t}$ **then**13:               Select the worst matching in set $\left\{\left(i^{t},M^{t}\left(i^{t}\right),M^{t}\left(M^{t}\left(i^{t}\right)\right)\right)\right\}$;14:               $M^{t}=M^{t}\setminus worst\left\{\left(i^{t},M^{t}\left(i^{t}\right),M^{t}\left(M^{t}\left(i^{t}\right)\right)\right)\right\}$;15:               Set flag=1;16:           **end if**17:           **if** $N(M^{t},j^{\prime t})==1$ **then**18:               $M^{t}=M^{t}\setminus \left(*,j^{\prime t},M^{t}\left(j^{\prime t}\right)\right)$;19:               Set flag=1;20:           **end if**21:           $M^{t}=M^{t}\cup \left(i^{t},j^{\prime t},k^{\prime t}\right)$;22:      **end if**23:   **end for**24:**end while**25:**return** $M^{t}$;

## 5. Performance Evaluation

In this section, we first construct a satellite constellation scenario by leveraging specialized network simulation tools. Subsequently, extensive simulation experiments are carried out to validate the performance metrics. Finally, the experimental results are compared against those of existing scheduling algorithms and analyzed in conjunction from multiple perspectives.

### 5.1. Simulation Setup

To verify the feasibility and availability of the proposed algorithm, an LEO satellite constellation scenario integrated with both wired and wireless links is constructed. The specific simulation tools utilized include Satellite Tool Kit (STK) 12.2.0 [18] and PyCharm 2024.1.3. STK is employed to establish the satellite constellation scenario. The detailed orbital parameters are derived from the Starlink constellation. Subsequently, the scheduling algorithms are implemented in Python3 utilizing the configuration parameters exported from STK. Specifically, the primary parameters adopted for the simulation are elaborated in Table 2, which were established according to references [10,16]. The number of TSN-enabled terminals within each satellite is configured as 5–8 [10]. To enable deterministic transmission, both terminals and switches within satellites are equipped with TAS capability. The wired and wireless integrated gateway is compatible with both TAS and TDMA. The TDMA is used for transmitting traffic over inter-satellite wireless links. The transmission rate of the intra-satellite wired link is configured as a constant value 1 Gbps. To accurately support time slot scheduling in the satellite network, the synchronization accuracy between devices is guaranteed through the IEEE 802.1AS protocol [21]. During the simulation experiments, the time-sensitive traffic is produced in a periodic manner. To avoid traffic backlog and mitigate the risk of congestion, the delay requirement is aligned with the traffic generation period.

### 5.2. Performance Analysis

This subsection concentrates on presenting and analyzing the performance metrics of various scheduling algorithms. To evaluate the effectiveness and efficiency of the proposed algorithm, comparative analyses are conducted against existing representative TAS scheduling algorithms. Specifically, Array Theory (AT) [22], Iterative-Optimization Modulo Theories (I-OMT) [23], and List Scheduling Per Link (LS-PL) [24] are configured as the comparison algorithms. In accordance with the specifications of the IEEE 802.1Qcc standard [25], we employ the shortest path routing algorithm to establish the routing path for each time-sensitive flow in the performance evaluation experiments.

#### 5.2.1. Delay Performance Comparison

**Figure 6:**
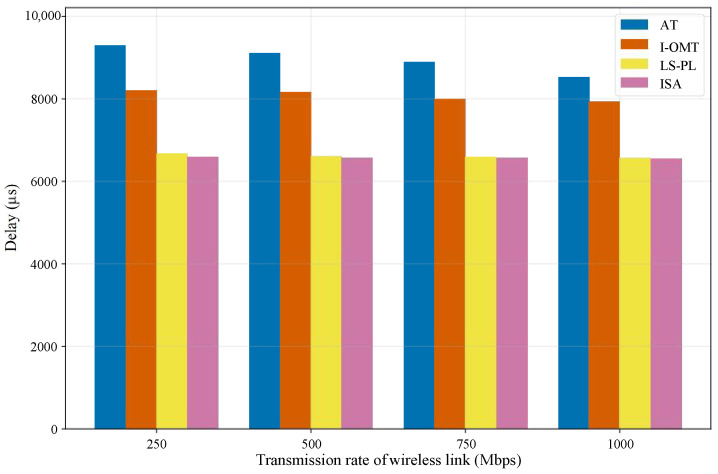
Average end-to-end delay under varied wireless rates.

A comparative analysis of average end-to-end delay across different algorithms with changing wireless link transmission rates is presented in Figure 6. The values in Figure 6 are derived from a custom-designed simulation dataset for wired and wireless integrated satellite networks. To ensure experimental validity, the dataset was developed in accordance with IEEE 802.1Qcc and IEEE 802.1Qbv standards. Regarding the network topology setup, the intra-satellite wired topology adopts a hierarchical tree structure, while the inter-satellite wireless topology uses a mesh topology. The number of TSN-enabled wired terminals within each satellite is configured as 5–8. For traffic configuration, a total of 20 periodic time-sensitive flows are randomly generated. The frame size of each flow is randomly selected from the range of 1000–1500 bytes. The transmission period of each flow is randomly chosen from the range of 10–15 ms. All time-sensitive flows are transmitted bidirectionally between wired and wireless nodes. To ensure statistical robustness, we conduct 100 independent experiments for each algorithm across each transmission rate scenario. In the subsequent step, the end-to-end delay is measured by tracking the duration from the transmission of the initial bit of a frame at the source node to the successful reception of the final bit at the destination node. Ultimately, the average end-to-end delay across all flows is calculated.

The experimental results demonstrate that the delay of all algorithms exhibits a downward trend as the transmission rate increases. This is attributed to the fact that higher transmission rates lead to reduced transmission delay, which in turn decreases the end-to-end delay. The delay results of the four algorithms exhibit a distinct three-gradient hierarchy: AT belongs to the high-delay group, I-OMT to the medium-delay group, and LS-PL and ISA to the low-delay group. When the transmission rate rises from 250 Mbps to 1000 Mbps, the delay of AT decreases by roughly 8.3%, whereas that of the I-OMT experiences a reduction of around 3.4%. As evidenced by the results, the delay of AT and I-OMT decreases significantly; this phenomenon reveals that transmission rate exerts a substantial impact on their delay performance. In contrast, the delay variations of LS-PL and ISA across the full rate range are approximately 1.4% and 0.5%, respectively. This suggests that LS-PL and ISA possess superior robustness in the face of dynamic wireless link transmission rate. Nevertheless, ISA achieves a lower delay compared to LS-PL. In brief, the proposed ISA algorithm effectively supports deterministic communication with strict delay requirements.

#### 5.2.2. Offset Performance Comparison

**Figure 7:**
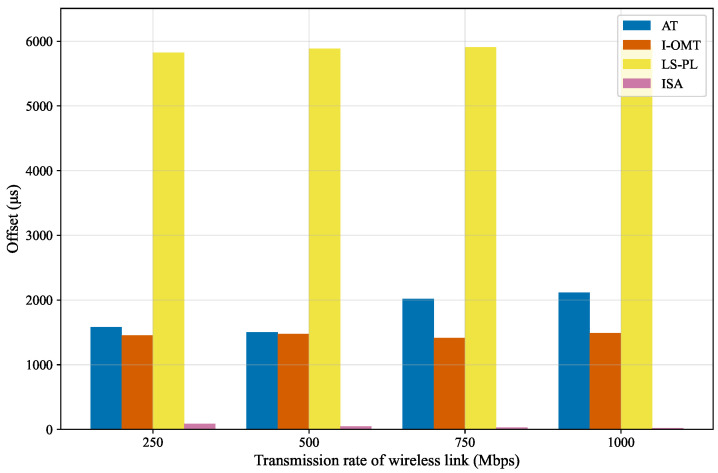
Offset under varied wireless rates.

The offset of different algorithms across varying wireless link transmission rates is depicted in Figure 7. Within the scope of deterministic communication, the offset refers to the release time of time-sensitive traffic when it is dispatched from the source node. Among all evaluated algorithms, LS-PL achieves the highest offset, which is several times higher than that of the other algorithms. This is attributed to the fact that the calculation of the time offset adopts a reverse-order mechanism in LS-PL. Under the premise of strictly satisfying the deadline constraints of time-sensitive traffic, LS-PL intentionally transmits data packets as late as possible. Other algorithms determine the time offset following a forward-order mechanism. Therefore, LS-PL exhibits a significantly higher time offset compared to the other algorithms. Additionally, the time offset of LS-PL exhibits a slight upward trend with the increase in wireless link transmission rate. This is because a higher transmission rate corresponds to a larger time offset in LS-PL under the same deadline constraints. These observations reveal that LS-PL not only has extremely poor time offset management capability but also shows disadvantages in rate adaptability. The time offsets of AT and I-OMT are observed to fall within the intermediate range, with values significantly lower than those of LS-PL but considerably higher than those of ISA. Specifically, the time offset of I-OMT remains stable across varying transmission rates, while that of AT exhibits an inconsistent trend, resulting in weaker rate adaptability compared to I-OMT. The experimental results also show that ISA obtains the minimum time offset across all specified transmission rates. ISA enables time-sensitive traffic to be released from the source node as soon as possible, thereby reducing the resource occupancy time. Moreover, its performance does not exhibit significant fluctuations with variations in transmission rate, indicating excellent rate adaptability and stability.

#### 5.2.3. Runtime Performance Comparison

**Figure 8:**
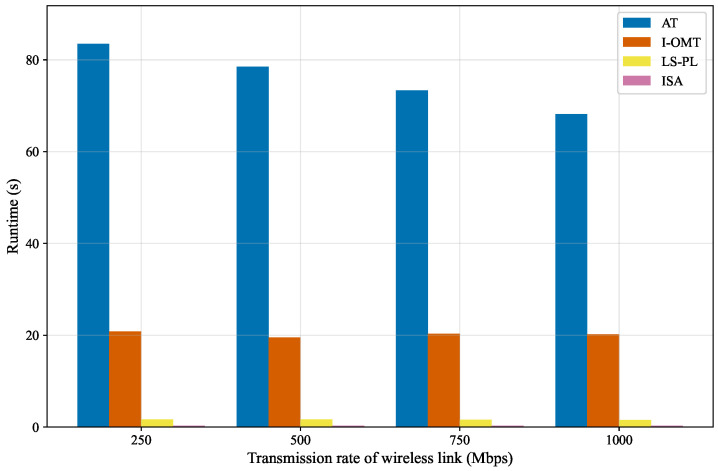
Runtime under varied wireless rates.

As presented in Figure 8, the runtime of each algorithm is measured and compared under different wireless link transmission rates. In the context of this experiment, the runtime denotes the time consumed by the algorithm to converge to a valid scheduling solution that meets the predefined constraints. Subsequently, multi-dimensional comparisons are conducted to clarify the performance differences among these algorithms. In terms of time efficiency, AT is the most time-consuming algorithm. As the transmission rate increases from 250 Mbps to 1000 Mbps, the runtime of AT decreases from approximately 83 s to 68 s. This is because AT dynamically adjusts the scheduling window size by incrementally adding transmission time in accordance with practical transmission requirements. Consequently, a higher transmission rate enables faster adjustment to an appropriate window size, thereby shortening the runtime. In contrast, the runtime of I-OMT remains stable at around 20 s across all tested transmission rates, indicating that the runtime consumption of AT is three to four times that of I-OMT. Notably, the runtime overhead of AT exceeds that of both LS-PL and ISA by tens of times. The higher runtime of AT stems from its array theory-based modeling approach, which generates a constraint system that encounters combinatorial explosion when handling scheduling problems. Specifically, AT constructs a three-dimensional nested dictionary for its decision variables. Meanwhile, the combination of these high-dimensional decision variables and associated scheduling constraints further exacerbates the runtime. Regarding stability, the runtime fluctuation of AT with transmission rates reaches approximately 15 s, reflecting poor stability. In comparison, I-OMT exhibits strong stability, with a runtime fluctuation of less than 2 s across the entire rate range. Meanwhile, the runtimes of LS-PL and ISA exhibit almost no variation with changing transmission rates, demonstrating excellent robustness. From the perspective of rate sensitivity, the runtime of AT presents a slow downward trend with increasing transmission rates but with significant fluctuations, indicating high sensitivity to rate changes. The runtime of I-OMT is minimally affected by rate variations, while the runtimes of LS-PL and ISA are completely insensitive to rate fluctuations. Overall, LS-PL and ISA demonstrate significant advantages in both time efficiency and stability, making them highly suitable for time-sensitive missions; however, ISA has a shorter runtime compared to LS-PL, giving it a minor advantage in computational efficiency.

#### 5.2.4. Memory Performance Comparison

**Figure 9:**
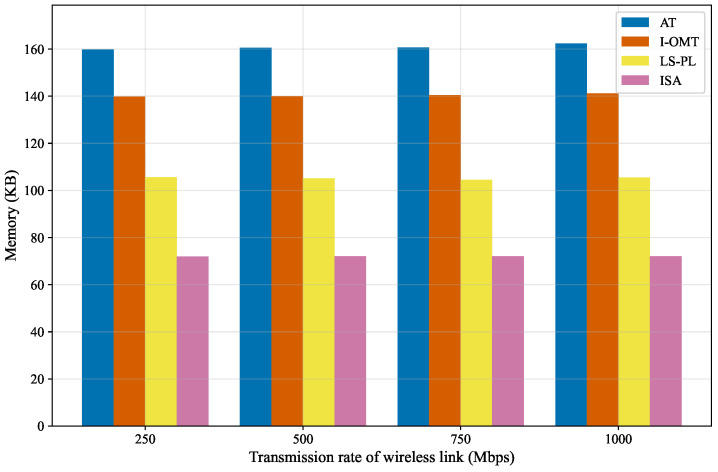
Memory consumption under varied wireless rates.

The memory consumption of different algorithms under varied wireless link transmission rates is illustrated in Figure 9. The experimental results indicate that the algorithms do not exhibit significant fluctuations in memory consumption as the wireless link transmission rate increases. More precisely, across all tested scenarios, the memory consumption of AT stabilizes at approximately 160 KB, I-OMT at around 140 KB, LS-PL at roughly 105 KB, and ISA at about 72 KB. These numerical observations demonstrate that the memory consumption of each algorithm remains stable despite variations in wireless link transmission rates. Through a comparative analysis of memory consumption among all algorithms, AT incurs the highest memory consumption; compared to AT, I-OMT achieves a 12.5% reduction in overhead, while LS-PL further reduces it by approximately 25% relative to I-OMT. Notably, ISA achieves the lowest memory consumption, representing an additional 31.4% reduction relative to LS-PL. To summarize, ISA outperforms the others in memory resource consumption, making it more suitable for deployment in resource-constrained application scenarios.

## 6. Conclusions

We proposed an integrated scheduling algorithm that incorporates principles from matching game theory, aiming at the problem of unstable delay caused by differentiated transmission characteristics of intra-satellite wired links and inter-satellite wireless links. First, we establish an integrated scheduling model that effectively quantifies the impact of scheduling time slots on the delay experienced at the satellite. Then, the established model is transformed into a cyclic three-sided matching game model to tackle the integrated scheduling problem. Subsequently, we design an integrated scheduling algorithm to obtain the time slot allocation results. Extensive experiments demonstrate that the proposed algorithm successfully achieves deterministic delay for time-sensitive traffic. In future work, we aim to investigate deterministic scheduling in large-scale satellite networks under massive traffic conditions.

## Figures and Tables

**Table 1:** Main notation and descriptions.

Notations	Descriptions
*t*	Topology time slot *t*, where t∈[1,T]
T	Total number of topology time slots
$I^{t}$	Set of time-sensitive traffic in *t*
$J^{t}$	Set of wired time slots in *t*
$K^{t}$	Set of wireless time slots in *t*
$M^{t}$	Set of three-sided matching in *t*
$i^{t}$	Specific time-sensitive traffic in *t*, $i^{t}\in I^{t}$
$j^{t}$	Specific wired time slot in *t*, $j^{t}\in J^{t}$
$k^{t}$	Specific wireless time slot in *t*, $k^{t}\in K^{t}$
$x_{i,j}^{t}$	Indicates whether $i^{t}$ is scheduled in $j^{t}$
$y_{j,k}^{t}$	Indicates whether $j^{t}$ is scheduled in $k^{t}$
$T_{i^{t}}$	Generation period of $i^{t}$
$B_{i^{t}}$	Data volume of $i^{t}$
$m_{i^{t}}$	The *m*th subperiod of $i^{t}$
$L_{w}$	Length of wired scheduling time slot
$L_{wl}$	Length of wireless scheduling time slot
$P_{i^{t}},P_{j^{t}},P_{k^{t}}$	The preference list of $i^{t}$, $j^{t}$, and $k^{t}$, respectively
$M^{t}\left(i^{t}\right),M^{t}\left(j^{t}\right),M^{t}\left(k^{t}\right)$	The preference set of $i^{t}$, $j^{t}$, and $k^{t}$, respectively

**Table 2:** Simulation parameters.

Parameter	Value
Altitude of the orbit	550 km
Inclination of orbit	53 deg
Number of orbits	14
Number of satellites per orbit	22
Total number of satellites in constellation	308
Number of inter-orbit ISLs per satellite	4
Rate of intra-satellite wired link	1 Gbps
Rate of inter-satellite wireless link	[250, 500, 750, 1000] Mbps
Number of terminals inside each satellite	5–8

## Data Availability

Data is contained within the article.

## Abbreviations

The following abbreviations are used in this manuscript:

STINs	Space–Terrestrial Integrated Networks
LEO	Low-Earth Orbit
TSN	Time-Sensitive Networking
DetNet	Deterministic Networking
FPGA	Field-Programmable Gate Array
TAS	Time-Aware Shaper
TDMA	Time-Division Multiple Access
ISA	Integrated Scheduling Algorithm
STK	Satellite Tool Kit
TST	Time-Sensitive Traffic

## References

[1] Xiao Z., Yang J., Mao T., Xu C., Zhang R., Han Z., Xia X.G. (2024). LEO Satellite Access Network (LEO-SAN) Toward 6G: Challenges and Approaches. IEEE Wirel. Commun..

[2] Xie H., Zhan Y., Zeng G., Pan X. (2021). LEO Mega-Constellations for 6G Global Coverage: Challenges and Opportunities. IEEE Access.

[3] Messenger J.L. (2018). Time-sensitive networking: An introduction. IEEE Commun. Stand. Mag..

[4] Nasrallah A., Thyagaturu A.S., Alharbi Z., Wang C., Shao X., Reisslein M., ElBakoury H. (2019). Ultra-Low Latency (ULL) Networks: The IEEE TSN and IETF DetNet Standards and Related 5G ULL Research. IEEE Commun. Surv. Tutor..

[5] Tian W., Gu C., Guo M., He S., Kang J., Niyato D., Chen J. (2024). Large-Scale Deterministic Networks: Architecture, Enabling Technologies, Case Study, and Future Directions. IEEE Netw..

[6] Sisinni E., Saifullah A., Han S., Jennehag U., Gidlund M. (2018). Industrial internet of things: Challenges, opportunities, and directions. IEEE Trans. Ind. Inform..

[7] Zanbouri K., Noor-A-Rahim M., John J., Sreenan C.J., Poor H.V., Pesch D. (2025). A Comprehensive Survey of Wireless Time-Sensitive Networking (TSN): Architecture, Technologies, Applications, and Open Issues. IEEE Commun. Surv. Tutor..

[8] Garcia-Rodriguez A., Lopez-Perez D., Galati-Giordano L., Geraci G. (2021). IEEE 802.11 be: Wi-Fi 7 strikes back. IEEE Commun. Mag..

[9] Seijo Ó., López-Fernández J.A., Val I. (2021). w-SHARP: Implementation of a high-performance wireless time-sensitive network for low latency and ultra-low cycle time industrial applications. IEEE Trans. Ind. Inform..

[10] Chaine P.J., Boyer M., Pagetti C., Wartel F. TSN support for quality of service in space. Proceedings of the 10th European Congress on Embedded Real Time Software and Systems.

[11] Sanchez-Garrido J., Aparicio B., Ramírez J.G., Rodriguez R., Melara M., Cercós L., Ros E., Diaz J. (2021). Implementation of a time-sensitive networking (TSN) Ethernet bus for microlaunchers. IEEE Trans. Aerosp. Electron. Syst..

[12] Peng G., Wang S., Li G., Huang T., Huang Y., Liu Y. (2025). Space-Terrestrial Integrated Time-Sensitive Networks: Architecture, Challenges, and Open Issues. IEEE Netw..

[13] Ma X., Li S., Guan Z., Li J., Sun H., Wang Y., Guo H. (2023). Time-Sensitive Networking Mechanism Aided by Multilevel Cyclic Queues in LEO Satellite Networks. Electronics.

[14] Wang F., Yao H., He W., Chang H., Xin X., Guo S. (2024). Time-Sensitive Scheduling Mechanism Based on End-to-End Collaborative Latency Tolerance for Low-Earth-Orbit Satellite Networks. IEEE Trans. Netw. Sci. Eng..

[15] Jiang X., Huang Y., Li J., He H., Chen S., Yang F., Yang J. (2023). Spatio-temporal routing, redundant coding and multipath scheduling for deterministic satellite network transmission. IEEE Trans. Commun..

[16] Peng G., Wang S., Huang T., Li F., Zhao K., Huang Y., Xiong Z. (2024). FastTS: Enabling Fault-Tolerant and Time-Sensitive Scheduling in Space-Terrestrial Integrated Networks. IEEE J. Sel. Areas Commun..

[17] (2016). IEEE Standard for Local and Metropolitan Area Networks—Bridges and Bridged Networks—Amendment 25: Enhancements for Scheduled Traffic.

[18] Han Z., Xu C., Zhao G., Wang S., Cheng K., Yu S. (2023). Time-varying topology model for dynamic routing in LEO satellite constellation networks. IEEE Trans. Veh. Technol..

[19] Stanescu D., Buzducea A. (2025). An Overview of Satellite Link Budget Sensitivity Based on Digital Modulation Schemes in Multi-Orbit Satellite Networks. Appl. Sci..

[20] Jia Z., Sheng M., Li J., Zhou D., Han Z. (2021). Joint HAP access and LEO satellite backhaul in 6G: Matching game-based approaches. IEEE J. Sel. Areas Commun..

[21] (2025). IEEE Standard for Local and Metropolitan Area Networks–Timing and Synchronization for Time-Sensitive Applications.

[22] Serna Oliver R., Craciunas S.S., Steiner W. (2018). IEEE 802.1Qbv Gate Control List Synthesis Using Array Theory Encoding. 2018 IEEE Real-Time and Embedded Technology and Applications Symposium (RTAS).

[23] Jin X., Xia C., Guan N., Xu C., Li D., Yin Y., Zeng P. (2020). Real-Time Scheduling of Massive Data in Time Sensitive Networks with a Limited Number of Schedule Entries. IEEE Access.

[24] Bujosa D., Ashjaei M., Papadopoulos A.V., Nolte T., Proenza J. (2022). HERMES: Heuristic multi-queue scheduler for TSN time-triggered traffic with zero reception jitter capabilities. Proceedings of the 30th International Conference on Real-Time Networks and Systems, Paris, France, 7–8 June 2022.

[25] (2021). IEEE/ISO/IEC International Standard-Telecommunications and Exchange Between Information Technology Systems—Requirements for Local and Metropolitan Area Networks—Part 1Q: Bridges and Bridged Networks AMENDMENT 31: Stream Reservation Protocol (SRP) Enhancements and Performance Improvements.

